# Hysteresis-free and dynamically resilient strain sensor enabled by interfacial coordination

**DOI:** 10.1126/sciadv.aea2450

**Published:** 2026-01-01

**Authors:** Jiang He, Jiaoya Huang, Rongrong Li, Ziyu Chen, Zemin Li, Runhui Zhou, Siyuan Wang, Wenchao Gao, Chuan Fei Guo, Rongrong Bao, Caofeng Pan

**Affiliations:** ^1^ Institute of Atomic Manufacturing, Beihang University, Beijing 100191, P. R. China.; ^2^ Department of Materials Science and Engineering, Southern University of Science and Technology, Shenzhen 518055, China.; ^3^ Beijing Institute of Nanoenergy and Nanosystems, Chinese Academy of Sciences, Beijing 101400, P. R. China.; ^4^ School of Physical Science, University of Chinese Academy of Sciences, Beijing 101408, China.

## Abstract

Mechanical hysteresis in soft materials remains a fundamental barrier to achieving accurate, high-speed strain sensing, especially under large and dynamic deformation. Here, we report a hysteresis-free strain sensor enabled by an interfacial coordination strategy, which integrates intrinsically stretchable dual-network universal bonding materials to establish robust adhesion between hyperelastic and hydrogel-dielectric hybrid systems. This architecture simultaneously enhances the elastic rebound stiffness of the composite and suppresses interfacial slippage, leading to a notable reduction in system-level hysteresis. A strain rate–dependent evaluation framework is proposed to systematically quantify dynamic hysteresis variability. The resulting sensor exhibits outstanding performance under extreme mechanical conditions, including 100% peak strain and strain rates up to 50% per second, maintaining a failure range below 1%. Moreover, the sensor demonstrates high linearity [coefficient of determination (*R*^2^) = 0.9998], an extended sensing range exceeding 200%, and superior mechanical durability. This work provides a comprehensive strategy toward hysteresis-free and dynamically accurate soft strain sensors, paving the way for next-generation human-machine interfaces and wearable electronics.

## Teaser

A hysteresis-free strain sensor enhances dynamic performance under extreme conditions, revolutionizing soft sensing technology.

## INTRODUCTION

Flexible sensors, as emerging electronic devices capable of perceiving mechanical deformation or stress, demonstrate promising application prospects across multiple domains, including medical monitoring, intelligent robotics (*1*–*7*), human-machine interfaces (*8*, *9*), smart textiles (*10*–*12*), motion healthcare (*13*–*15*), and industrial infrastructure monitoring, owing to their exceptional sensitivity (*14*, *16*–*26*), stretchability (*27*–*34*), and conformability to complex surfaces (*35*–*40*). An ideal strain sensor requires exceptional measurement accuracy and superior signal consistency under both static and dynamic operating conditions. Nevertheless, the current hysteresis calculation for the strain sensor is based on the loading-unloading curves of materials measured under quasistatic material conditions (fixed strain magnitude and strain rate). The sensor signal errors caused by material hysteresis in real-world dynamic application scenarios cannot be accurately quantified using this method. The intrinsic time-dependent viscoelastic behavior of elastomeric substrates and irreversible slippage of the heterogeneous interface may induce pronounced signal hysteresis (*41*–*44*) when subjected to large-strain or high–strain-rate deformations, thereby generating signal acquisition blind spots. Consequently, conventional flexible strain sensors frequently exhibit a substantial discrepancy in dynamic performance metrics when compared to their quasistatic counterparts. This inherent limitation in temporal response characteristics could compromise the reliability of sensing systems using such devices, potentially leading to erroneous triggering or safety-critical system malfunctions in practical implementations.

Capacitive soft strain sensors are highly suitable for practical applications due to their excellent linearity and stretchability (*45*–*52*). High-stretchable electrode materials and dielectric layers are fundamental materials for constructing capacitive strain sensors. Currently, research on stretchable electrode materials primarily focuses on achieving this by blending high-modulus conductive materials (such as carbon and metals) with elastic rubber materials (*51*, *53*–*55*) or through composite structural interfaces (*27*, *28*, *56*–*58*). However, because of the notable differences in modulus between these materials, interfacial frictional slipping inevitably occurs during deformation, leading to energy dissipation and the emergence of mechanical hysteresis (*43*). In contrast, hydrogels exhibit excellent stretchability, with a wide tunable modulus range (from kilopascals to megapascals), and have intrinsic ionic conductivity, making it unnecessary to add conductive fillers to achieve satisfactory conductivity. These properties enable hydrogels to substantially reduce hysteresis due to modulus differences while meeting the essential requirements for both stretchability and conductivity in our devices. Although under ideal testing conditions without external interference, there is no difference in the sensitivity test results between devices constructed with low–dielectric-constant materials and those with high–dielectric-constant materials. However, devices using low–dielectric-constant materials exhibit extremely low initial capacitance, making their signals highly susceptible to interference from external electromagnetic signals and the inherent noise of testing equipment. More critically, capacitive strain sensors tend to couple with external objects (such as metal conductors, the human body, and the ground). These coupling signals can easily overwhelm the sensing signals, resulting in an extremely low signal-to-noise ratio of the device, rendering it impractical for real-world applications. On the basis of the anti-interference requirements of sensors in practical applications and the operational principles of capacitive soft strain sensors, an ideal dielectric elastomer for high-performance sensors should exhibit a sufficiently high dielectric constant, large elastic strains, and low hysteresis (*5*, *46*, *53*, *57*). However, the irreconcilable conflict between the viscoelastic mechanical properties and electrical properties of soft materials restricts their applications in devices subjected to large strains and high-speed dynamic conditions. The magnitude of the chain segmental friction in elastomers mainly governs their hysteresis characteristics (*59*–*62*). Low-polarity symmetric molecular structures can effectively reduce intermolecular chain interactions, thereby decreasing frictional dissipation and hysteresis. Natural rubber and silicone elastomers exhibit nearly ideal spring hyperelastic behavior and can be appropriately modeled using a Hooke model with an elastic modulus *k*. Nevertheless, these architectures inherently have low dielectric constants, making them unsuitable for dielectric applications. For elastomers that require high dielectric constants [such as poly(vinylidene fluoride-co-trifluoroethylene) copolymer) (*63*, *64*), polyacrylate, (*65*)] or ionic conductivity (such as hydrogel), polymers with high-polarity asymmetric molecular structures are usually used. Their strong intermolecular interactions generate substantial frictional energy dissipation during deformation, resulting in pronounced hysteresis behavior. Therefore, to construct an ideal strain sensor with both static and dynamic performance, the development of low-hysteresis composite material preparation technology is urgently required.

This study presents a hysteresis-mitigated strain sensing system achieved through an interface coordination strategy. Guided by the Kelvin-Voigt viscoelastic paradigm of elastomeric matrices, a rational interfacial synergistic architecture was conceptualized to enhance the rebound stiffness through viscoelastic energy modulation, thereby achieving substantial hysteresis suppression. The implementation of this architecture necessitated the development of a dual-network deformable adhesive system that demonstrated molecular-scale hooking-interlocking mechanisms. This innovative adhesive enables robust interfacial integration between heterogeneous elastomers and hydrogel polymer networks while preserving the intrinsic stretchability. The resultant interface engineering facilitated the assembly of low-hysteresis elastomeric composites with heterogeneous multilayer configurations using adaptable adhesive integration techniques. A prototype capacitive strain transducer with an optimized multilayer architecture demonstrated linear response characteristics, achieving high linearity [coefficient of determination (*R*^2^) = 0.9998] across a 200% wide strain range. This study established a strain-rate–dependent dynamic hysteresis evaluation framework for strain sensors. Through systematic experimental acquisition of the sensor’s working range data under multigradient strain rates, the constructed mathematical calculation method enables dynamic evaluation of the reliability of randomly fluctuating strain rate and amplitude data at any given time point, achieving real-time dynamic quantification of the sensor hysteresis magnitude, providing a theoretical framework and technical pathway for researching sensor dynamic characteristics. A low-hysteresis strain sensor with a sub-1% dynamic failure range (100% strain, 50% s^−1^ strain rate) was successfully prepared. Under extreme mechanical dynamic testing conditions (100% peak strain, 1000% s^−1^ strain rate), the low-hysteresis sensor maintains a sub-5% failure range, whereas high-hysteresis devices exceeded the 40% failure range. The tough bonded architecture further endowed the system with enhanced mechanical resilience, as evidenced by 93.56% interdevice consistency in performance metrics and superior damage tolerance. The optimized dynamic response characteristics enable notable latency reduction in human-machine interface applications. Robotic validation trials demonstrated a marked improvement in task scores during high-dynamic manipulation sequences compared to conventional sensing platforms, confirming the operational reliability of the system in stochastic dynamic environments.

## RESULTS

### Design and principles for hysteresis-free sensing

**Fig. 1.**
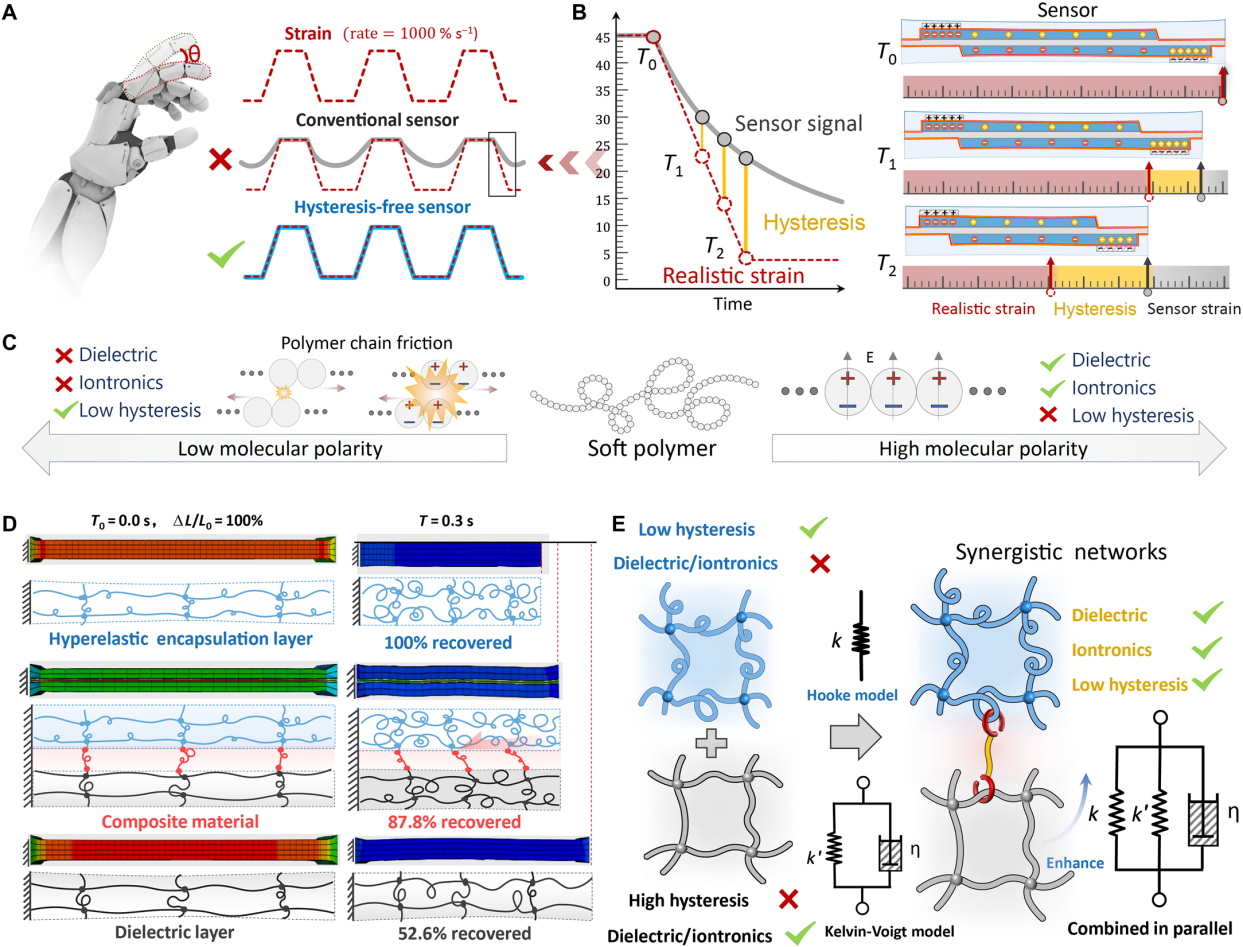
Design and principles for hysteresis-free sensing. (**A**) In human-machine collaborative systems, conventional strain sensors generate hysteresis signals and cause blind zones when subject to a dynamic strain, while a hysteresis-free strain sensor generates nonhysteresis signals for ultrahigh strain rate dynamic sensing. (**B**) The strain sensors constructed from viscoelastic materials exhibit notable mechanical hysteresis during dynamic deformation, manifested as a phase delay of the sensing signal relative to the actual strain, thereby inducing time-domain distortion in the output signal. (**C**) The irreconcilable conflict between the dynamic mechanical properties (low hysteresis) and electrical properties (dielectric and iontronics) of soft materials. The rational design principles (**D**) and corresponding theoretical model (**E**) elucidate the operational mechanisms of multifunctional heterogeneous elastomeric composites that achieve hysteresis-free strain measurement performance through interfacial synergistic architecture.

The idealized strain-sensing unit should have dynamic tracking capability for real-time synchronization with actual strain variations, thereby enabling high-fidelity measurements of transient strain fields (Fig. 1A). However, constrained by the intrinsic viscoelastic properties inherent in soft matter materials, a prevalent strain phase hysteresis phenomenon occurs during sensor dynamic response (Fig. 1B). This lagging effect induces substantial measurement deviations in transient strain values, ultimately leading to functional failure of the device.

Regarding performance optimization of capacitive strain sensors, the core challenge resides in the design and synthesis of high-performance dielectric/iontronic electroactive elastomeric composites. Research demonstrates that traditional elastomeric polymer systems exhibit pronounced performance antagonism between low-hysteresis characteristics and dielectric/iontronic properties (Fig. 1C). This inherent contradiction, originating from the material relaxation mechanisms and molecular chain conformational changes, substantially restricts the comprehensive performance enhancement of the devices. In general, the magnitude of the chain segmental internal friction within elastomeric materials exerts a predominant influence on their hysteretic characteristics. Molecular architectures characterized by low-polar symmetric configurations demonstrate particular efficacy in diminishing intermolecular chain interactions, thereby conducive to attenuating frictional dissipation and hysteresis phenomena. Representative exemplars, including natural rubber and silicone elastomers, exhibit hyperelastic behavior approximating ideal spring mechanics, appropriately modeled through a Hooke constitutive relationship with elastic modulus *k*. Nevertheless, these structural configurations inherently manifest limited dielectric permittivity, rendering them suboptimal for dielectric applications. Conversely, elastomeric systems requiring elevated dielectric constants typically incorporate polyacrylate-based polymers with highly polar asymmetric molecular architectures. These materials inherently have intensified intermolecular forces that engender substantial energy dissipation through frictional losses during deformation cycles, consequently inducing a pronounced hysteresis behavior. The strain recovery dynamics in such viscoelastic systems are aptly characterized by a Kelvin-Voigt model comprising parallel-connected elements: an elastic spring with modulus *k*′ and a viscous damper with coefficient η. During the strain recovery phase, the spring element facilitates restorative potential energy release, while the damper component exerts velocity-proportional resistance, collectively producing time-dependent retardation in dimensional restitution and resultant hysteresis loop formation.

In this study, we propose an interfacial synergistic architecture in which a superelastic layer is integrated with dielectric/iontronic layers through interfacial bonding. The design framework positions the superelastic component as an ideal spring element in parallel connection with the dielectric layer’s Kelvin-Voigt viscoelastic model. This mechanical coupling configuration effectively enhances the system’s rebound stiffness, thereby substantially mitigating hysteresis phenomena. As shown in Fig. 1D, finite element modeling was used to simulate the dynamic recovery behavior of the composite system. This system comprised two layers of low-hysteresis silicone elastomer (Ecoflex) from Smooth-On as the encapsulation layer and a sandwich of commercial 3 M Very High Bond (VHB) polyacrylate tape as the dielectric layer. VHB was selected as the dielectric elastomer because of its excellent elasticity and dielectric properties, which have led to its widespread use in applications such as flexible sensors (*66*) and artificial muscles (*67*). The simulation incorporated a seamlessly bonded Ecoflex-VHB interface with optimized adhesion parameters, explicitly excluding the interfacial energy dissipation mechanisms. Simulation data revealed that this synergistic strategy substantially improved the dielectric elastomer’s recovery rate, achieving 87.8% strain recovery at 0.3 s compared to the baseline of 52.6%. Building upon these findings, the development of advanced interfacial engineering protocols becomes crucial for establishing parallel mechanical coupling between superelastic and viscoelastic networks (Fig. 1E). Critical to this approach is the implementation of low-loss strain transmission pathways within the composite network, enabling coordinated deformation synchronization across heterogeneous components. Through this methodology, functional devices exhibiting near-ideal dynamic response characteristics may be fabricated.

### Fabrication and characterization of the hydrogels’ and elastomers’ tough-bonding composite

**Fig. 2.**
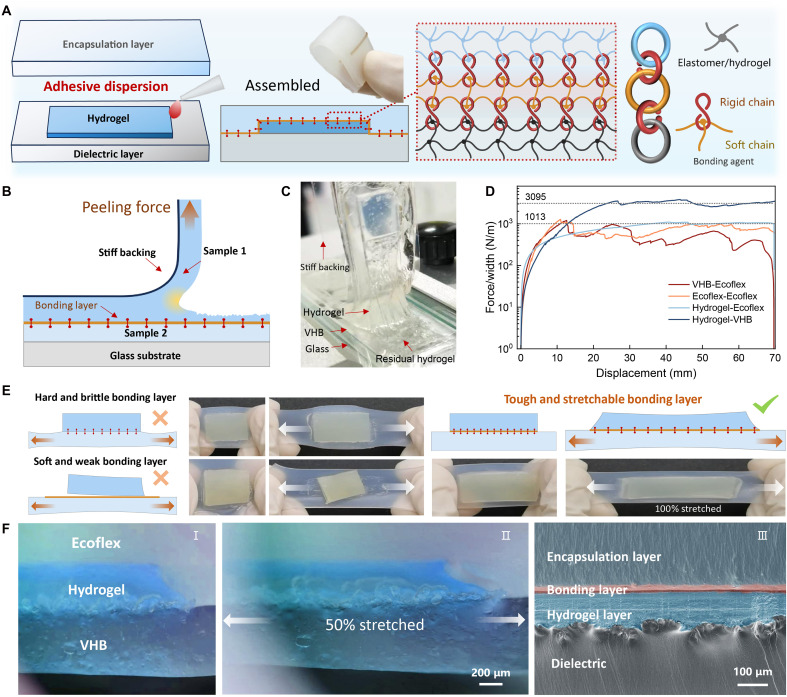
Fabrication and characterization of the hydrogels’ and elastomers’ tough-bonding composite. (**A**) Schematic illustration of the bonding process of hydrogels, dielectric layer, and encapsulation layer. A dual-network stretchable adhesive that has been developed is used to promptly and firmly bond different functional soft layers. The bonding agent connects different polymer networks by means of a molecular-level hooking-interlocking effect. As a result, the interface is both instantaneous and tough while still maintaining its stretchability. (**B**) Schematic illustration of the 90° peel strength test to evaluate the interfacial adhesion strength of adhesive bonds among three distinct materials: hydrogel, VHB, and Ecoflex. (**C**) Photos of the high-strength double-network hydrogel-VHB during the 90° peeling test. The crack propagates within the hydrogel, and the residual layer of hydrogel on the surface can be observed. (**D**) Peeling force per unit width measurements for hydrogel-elastomer hybrid bonding interfaces. (**E**) The demonstration of the strain transfer performance of the adhesive. The dual-network adhesive combines high interfacial strength with excellent stretchability, enabling efficient interlayer strain transfer. (**F**) The cross-sectional images of the multilayer hydrogel-elastomer bonding hybrids include photos and scanning electron microscopy (SEM) images. These images show the seamless microscopic integration of the materials.

Figure 2A demonstrates the multifunctional heterogeneous elastic composite structure constructed on the basis of a physical entanglement mechanism. This study developed a rigid-flexible dual-network stretchable adhesive with universal bonding capabilities, which primarily functions through a dual-network synergistic mechanism. The highly elastic adhesive network system comprises highly flexible silicone molecular chains, which exhibit the distinctive characteristic of achieving excellent deformation coupling with the low-hysteresis layer without imposing deformation constraints on the elastic substrate. Specifically, upon deformation of the low-hysteresis layer, the stress-transfer mechanism of the low-modulus adhesive network effectively induces synchronized transverse deformation in the high-hysteresis layer. This multilevel strain-transfer mechanism facilitates cooperative deformation behavior in the transverse dimension within the ternary system of low-hysteresis layer–adhesive interlayer–high-hysteresis layer. The rigid network is composed of polycyanoacrylate molecular chains, whose unique feature lies in the directional penetration and diffusion of cyanoacrylate monomers (*68*) into bonded materials (including hydrogels and elastomers) during adhesion, followed by in situ polymerization to form interpenetrating entangled networks (fig. S1). The polycyanoacrylate molecule contains a cyano group (-C≡N), whose high polarity induces strong dipole-dipole interactions between molecular chains. These interactions restrict the free movement of polymer chains, endowing them with high conformational stability while reducing segmental flexibility, thereby imparting pronounced rigidity to the polymer matrix. This rigid nature renders the formed physical entanglement structure resistant to dissociation, thereby enabling internetwork connections via a molecular-level hooking-interlocking mechanism. This physical entanglement-based bonding mechanism eliminates the reliance on material surface energy inherent in traditional chemical bonding, enabling simultaneous high-strength adhesion between low–surface-energy elastomers [e.g., polydimethylsiloxane (PDMS)] and high–surface-energy hydrogels [e.g., polyacrylamide (PAAm)].

We designed a 90° peel test to evaluate the interfacial adhesion strength of adhesive bonds among three distinct materials: hydrogel, VHB, and Ecoflex. The interfacial adhesion strength between adhesive samples 1 and 2 was assessed by stacking the samples as illustrated. A stiff backing, composed of a 20-mm-wide polyethylene terephthalate (PET) film, was affixed to the top surface to prevent elongation of sample 1 (10 mm wide, 2 mm thick, and 100 mm long) in the direction of the peel force. Concurrently, during the peeling test, sample 2 (20 mm wide and 70 mm long) was secured on a rigid glass substrate at the bottom. For the hydrogel-VHB test, sample 1 corresponds to the hydrogel and sample 2 to VHB. As depicted in Fig. 2C, during the challenging hydrogel-VHB 90° peel test, crack propagation occurred within the hydrogel rather than at the hydrogel-elastomer interface. This type of cohesive failure resulted in a residual layer of hydrogel remaining on the bonding surface, indicating that the adhesive strength at the interface exceeds the intrinsic strength of the hydrogel. However, because of the inferior mechanical properties of conventional hydrogels, this observation does not accurately reflect the actual interfacial strength. To address this limitation, we developed a high-strength double-network hydrogel. Experimental results indicate that the peel strength (peel force per unit width) for the weak hydrogel is 76 N/m, whereas the high-strength hydrogel exhibits a peel force of 3069 N/m (fig. S2). This value does not represent the ultimate strength of the adhesive; to ascertain a true measure, hydrogels with even greater strength must be synthesized. To further confirm the performance of our developed adhesive material, we compared its adhesion strength with that of commercially available adhesives. The data demonstrate a continued advantage for our material, with two commercial adhesives yielding peel forces of 61 N/m for Sil-Poxy and 1589 N/m for LOCTITE 406 (fig. S3). Using the same testing method, we also measured the interfacial adhesion strengths for VHB-Ecoflex, hydrogel-VHB, Ecoflex-Ecoflex, and hydrogel-Ecoflex samples. As shown in Fig. 2D, the peel forces for all samples exceed 1000 N/m, signifying that the bonding strength at the interface surpasses 1000 N/m.

In addition to requiring the adhesive layer to have high adhesion strength to ensure interfacial integrity, we also demand efficient strain transfer across the adhesive interface with minimal energy loss. This necessitates adhesive materials that simultaneously exhibit strong adhesion, low modulus, and high ductility. Here, we compare commercial single-network adhesives with our rigid-flexible dual-network adhesive (Fig. 2E). The left panel demonstrates that pure rigid-network adhesives, while providing substantial adhesion strength, exhibit excessively high interfacial modulus. This results in strain isolation at one side of the interface, hindering effective strain transmission. Flexible molecular networks, although stretchable at the interface, show weak adhesion to hydrogels, leading to interfacial separation under large tensile strains and subsequent device failure. To investigate the impact of adhesive component ratios on performance, we prepared a series of materials with varying compositions and evaluated their interfacial adhesion properties. Experimental results (fig. S4) revealed that at a mass ratio of polysiloxane:2,2,4-trimethylpentane:cyanoacrylate = 5:4:1, the adhesive exhibited optimal performance, achieving a balance of high elasticity and strong adhesion. In contrast, other formulations (3:4:3 and 4:4:2) induced two distinct failure modes: (i) interface debonding resulting from component incompatibility and (ii) nucleation and propagation of microcracks under tensile stress due to an excessive presence of the brittle phase. Both failure mechanisms contribute to a nonuniform strain field distribution within the interfacial region, leading to substantial energy dissipation at the interface. Our developed rigid-flexible dual-network adhesive combines low modulus with excellent stretchability, enabling efficient interlayer strain transfer while maintaining high interfacial strength. Even under substantial deformation, the interface remains stably bonded (right panel). Optical microscopy images (Fig. 2, F and G) confirm no interfacial delamination at 50% tensile strain. Smooth strain transmission across layers was observed without substantial strain isolation between different material layers. Scanning electron microscopy (SEM) cross-sectional images (Fig. 2H and fig. S5) reveal seamless microscopic integration of materials, with no visible interfacial separation even after repeated stretching cycles.

### Design and fabrication of the hysteresis-free strain sensor and its performance characterization

**Fig. 3.**
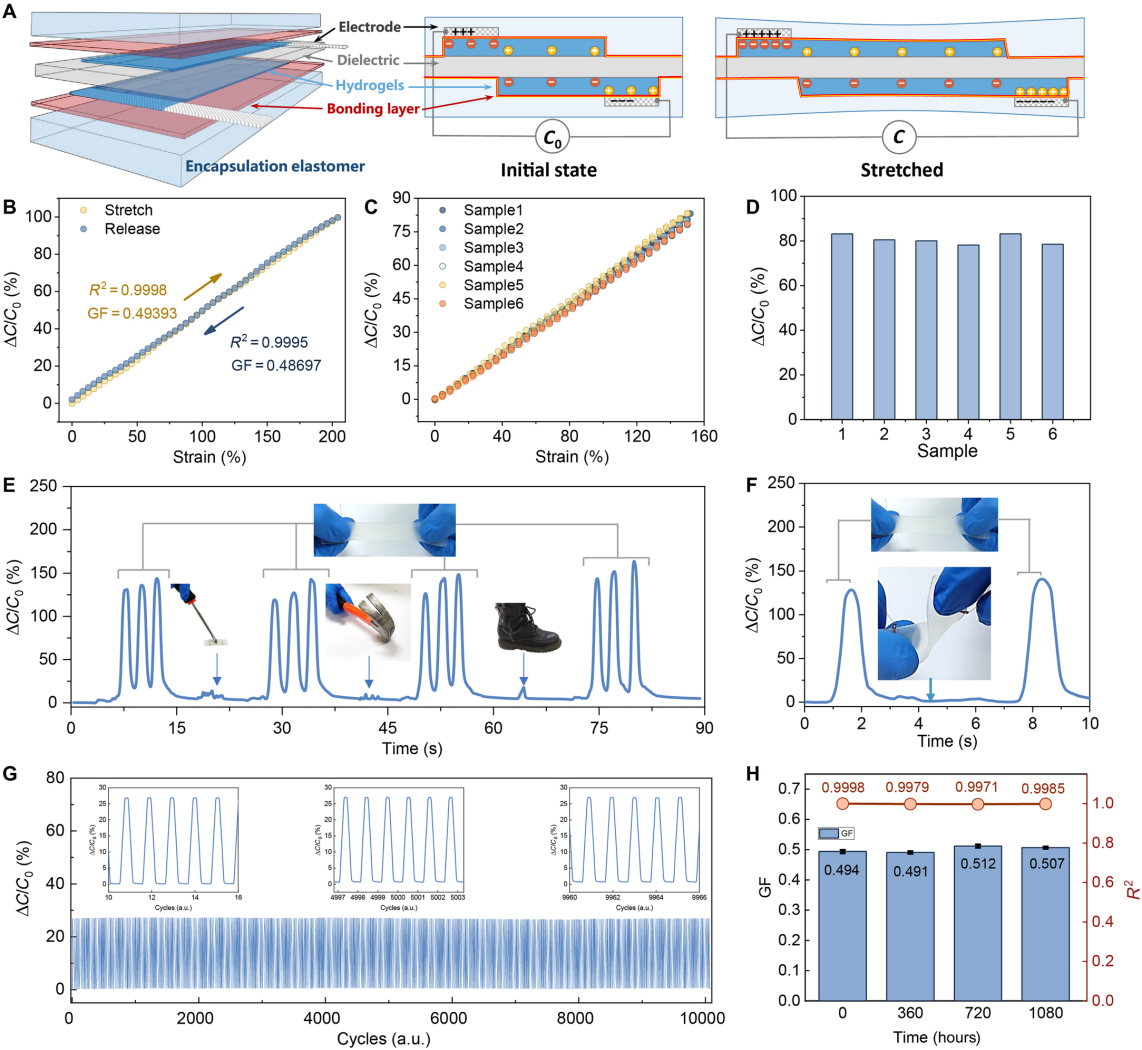
Structural configuration and integrated performance characterization of the hysteresis-free strain sensor. (**A**) Schematic illustration and operational mechanism of the multilayer flexible architecture underlying the capacitive strain-sensing platform. (**B**) Capacitive strain-response profiles under cyclic tensile loading and unloading regimes, demonstrating linear signal output across 0 to 204% applied strain. (**C**) and (**D**) are device-to-device reproducibility assessments across six sensor units subjected to 150% strain cycling. (**E**) The robustness of the sensor under normal stretching, stabbing from a screwdriver, striking from a hammer, trampling from a volunteer with 51 kg, and unwished twisting (**F**). (**G**) Long-term operational stability is quantified through 10,000 continuous strain cycles at 50% deformation amplitude. a.u., arbitrary units. (**H**) Temporal performance fluctuation analysis over an extended 1080-hour observation period. GF, gauge factor.

We constructed the hysteresis-free strain sensor via assembly and tough bonding of multifunctional soft layers. As shown on the left of Fig. 3A, the capacitive strain transducer architecture (Fig. 3A, left) was configured with VHB dielectric layers, patterned sodium hyaluronate PAAm (SH-PAAm) hydrogel electrodes, and Ecoflex/VHB encapsulation layers. On the basis of our bonding stratagem, the multilayered devices can be easily assembled by this stacking method: Initially, the conductive SH-PAAm hydrogel electrodes were fabricated via in situ polymerization and patterned on the front and back side of the VHB dielectric film (fig. S6). Subsequently, the Ecoflex/VHB encapsulation layers were bonded to the composite film by our dual-network adhesive, thus the layered structure strain sensor was fabricated.

This multilayered configuration facilitated exceptional linear response characteristics (*R*^2^ = 0.9998 under tensile loading, *R*^2^ = 0.99949 during relaxation) across 204% strain domains (Fig. 3B), with experimentally measured gauge factor (GF) of 0.487 showed excellent agreement with theoretical predictions. The hysteresis-free performance originated from synergistic interfacial adhesion between hydrogel-elastomer components and intrinsic mechanical compliance of constituent materials. Device reproducibility was achieved through patterning precision during hydrogel electrode fabrication, yielding interdevice signal variation below 6.44% at 150% strain (Fig. 3C) and minimum reproducibility thresholds exceeding 93.56% (Fig. 3D). Operational robustness evaluation revealed cryogenic resilience (−10°C; fig. S7) through lithium chloride (LiCl)/glycerol–modified hydrogel formulations, withstanding damage including puncture (screwdriver penetration), impact loading (hammer strikes), 51-kg human trampling (Fig. 3E), and multiaxis deformation (Fig. 3F). The sensors function during demanding conditions and maintain consistent behavior in all experiments, demonstrating their excellent mechanical robustness.

The fabricated sensors exhibited exceptional mechanical resilience and prolonged operational durability. As illustrated in Fig. 3G, the devices maintained consistent electromechanical performance through continuous cyclic deformation testing at 50% strain amplitude over 18 hours, with signal variation remaining within ±2% after 10,000 loading-unloading cycles. Long-term stability assessments indicated the sensing functionality under normal temperature and pressure. The results showed negligible performance changes after 1080 hours (Fig. 3H). This stability profile can be mechanistically attributed to the synergistic effects of the composite encapsulation architecture and the hydrogel matrix’s moisture-locking capabilities, where the SH-LiCl-glycerin ternary system effectively maintains interfacial hydration while suppressing water molecular migration. Specifically, Li^+^, as the smallest cation in the periodic table (with an ionic radius of ~76 pm), exhibits exceptionally high charge density and polarizability. When present in an aqueous system, Li^+^ forms stable ion-dipole bonds with the oxygen atoms of water molecules (the electronegative end of polar molecules) through strong electrostatic interactions (*69*). To further enhance the moisture retention capability of this system, we introduced glycerol as a synergistic component. The glycerol molecules engage in multiple hydrogen bonds with water molecules via their abundant hydroxyl (–OH) groups, notably increasing the binding energy of water within the gel network. This dual-component synergistic effect achieves a multilevel “locking” mechanism for free water molecules (*29*). In terms of engineering optimization, we also implemented elastic encapsulation technology, which effectively suppresses water evaporation kinetics by constructing a physical barrier layer. These collective findings substantiate the sensor’s excellent robustness and long-term operational reliability in practical applications.

### The design and principles of the hysteresis-free sensing structure

**Fig. 4.**
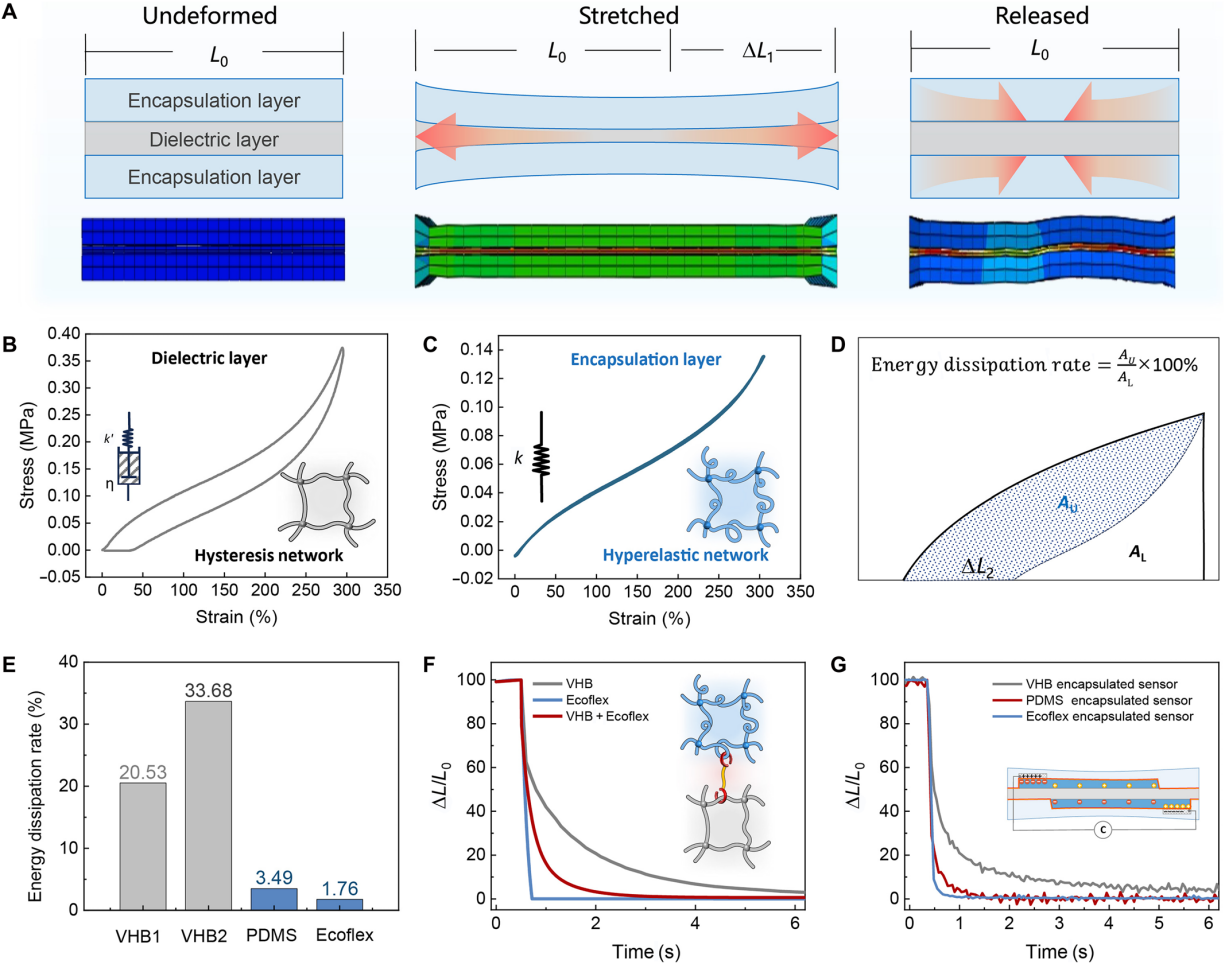
The design and principles of the hysteresis-free sensing structure. (**A**) Schematic and finite element analysis simulation for minimizing the hysteresis effect of the dielectric layer. (**B** and **C**) Representative stretching-relaxation curves for the viscoelasticity VHB elastomer (dielectric layer) and superelasticity Ecoflex elastomer (encapsulation layer). Energy loss calculation method (**D**) and the energy dissipation rate values (**E**) of VHB, PDMS, and Ecoflex. (**F**) The simulation results of the recovery displacement curves are presented for pure VHB, pure Ecoflex, and the VHB sandwiched by Ecoflex after 100% stretching and subsequent free releasing. (**G**) The response curves of three kinds of sensors, including the VHB encapsulated sensor, the PDMS encapsulated sensor, and the Ecoflex encapsulated sensor. The strain was released by the linear motor at the same rate of 50% s^−1^. The data on length recovery versus time is derived through reverse calculation from the corresponding sensor-measured electrical signals.

Hysteresis represents a critical challenge in achieving precise strain sensing performance, primarily attributed to the viscoelastic characteristics of dielectric materials. While substantial research efforts have been dedicated to developing low-latency sensing devices, the mitigation of hysteresis under large tensile deformations (exceeding 100% strain) and extreme strain rates remain inadequately addressed, posing notable limitations for real-time monitoring applications. This study proposes an interfacial engineering strategy to effectively suppress hysteresis through material hybridization, as systematically illustrated in the mechanism diagram (Fig. 4A). The encapsulation architecture uses hyperelastic elastomer layers that generate interfacial stress confinement during strain recovery, thereby counteracting the viscoelastic relaxation dynamics of the dielectric core and enhancing structural resilience.

In this work, we selected typical polyacrylate elastomers (VHB) and silicone elastomers (Ecoflex and PDMS) as representative research subjects. In our experiment, two types of polyacrylate elastomers were used. VHB1 refers to the 3M VHB 4905 product, while VHB2 refers to the 3M VHB 4920 product. VHB elastomers are widely used in flexible sensors and actuators for dielectric elastomer applications due to their high dielectric constant and excellent stretchability. However, they exhibit notable hysteresis during stress-strain sensing within specific frequency ranges, leading to signal distortion. In contrast, silicone elastomers demonstrate hyperelastic characteristics, although their relatively low dielectric constants limit their applications. To probe the hysteresis behaviors of different elastomers, cyclic tensile tests were used. As shown in Fig. 4B, the tensile-recovery curves of the VHB dielectric layer material revealed pronounced hysteresis loops, whereas the silicone elastomers exhibited typical hyperelastic behavior (Fig. 4C), showing minimal energy loss during cyclic testing. To quantify energy dissipation during cyclic loading, we calculated the energy dissipation rates for both materials (Fig. 4, D and E). The results indicate that silicone elastomers exhibit extremely low energy dissipation rates in single cycles (Ecoflex: 1.76%; PDMS: 3.49%), while polyacrylate elastomers demonstrate energy dissipation rates nearly an order of magnitude higher (VHB1: 20.53%; VHB2: 33.68%).

Finite element modeling was carried out to comparatively analyze the deformation recovery kinetics of three configurations: homogeneous VHB, homogeneous Ecoflex, and VHB-Ecoflex composite under 100% tensile strain (movie S1). The simulation results showed distinct recovery profiles. Pure Ecoflex had an instantaneous shape recovery (τ < 0.5 s) because of its entropy-driven hyperelastic response. In contrast, pure VHB showed pronounced viscoelastic retardation and required a recovery duration of more than 6 s (Fig. 4F). The hybrid VHB-Ecoflex configuration presented intermediate hysteresis characteristics (τ ≈ 2.8 s), indicating a synergistic interaction between the viscoelastic dissipation of VHB and the elastic restorative forces exerted by the Ecoflex encapsulation layers. These computational findings confirm that interfacial stress modulation through hyperelastic encapsulation can effectively reduce the overall system’s viscoelastic energy dissipation. This mechanical metamaterial approach offers a versatile strategy for developing high-precision strain sensors with minimized time-dependent signal drift. Encouraged by the simulation results, we developed a series of sensors encapsulated by polyacrylate elastomers and silicone elastomers. The strain response curves of the sensors are consistent with the above simulation calculations. The data revealed that the sensors encapsulated by low-hysteresis elastomers (Ecoflex and PDMS) had shorter latency (90% strain rebound in 0.869 s) compared to those encapsulated by high-hysteresis elastomers (VHB) (90% strain rebound in 2.211 s) (Fig. 4G). In addition, how the ratio of thicknesses between the different layers affects the hysteresis in the mechanical behavior of the whole stack has been investigated. As the encapsulation thickness increases, the overall hysteresis of the device decreases. However, when the ratio of encapsulation layer (Ecoflex) to dielectric layer (VHB) reaches 3.2:1, the change in hysteresis becomes nearly negligible (fig. S8).

### Dynamic hysteresis sensing performance

The phenomenon of hysteresis arises from the noninstantaneous relaxation kinetics of polymer chain reconfiguration processes inherent to viscoelastic materials. While standardized quantification methodologies for hysteresis remain undefined within current research paradigms, a prevalent analytical approach involves computing hysteresis magnitude through differential integration of loading-unloading pressure-response curves (fig. S9). Notably, hysteresis indices demonstrate notable parametric dependency on operational variables including strain excitation ranges and strain rate, thereby mandating rigorous standardization protocols for comparative assessments. Current characterization techniques predominantly use quasistatic loading-unloading protocols with constrained strain amplitudes and deformation rates for hysteresis quantification. However, this methodological constraint necessitates critical examination, as the temporal decoupling between quasistatic hysteresis parameters and dynamic operational scenarios introduces substantial challenges in compensating for signal phase discrepancies during transient measurements. The fundamental incongruity between quasistatic hysteresis metrics and time-dependent viscoelastic relaxation dynamics ultimately compromises the accuracy of error quantification and correction algorithms for dynamic sensor signals. This critical gap in metrological standardization underscores the imperative for establishing a unified evaluation framework incorporating dynamic hysteresis quantification metrics to enable rigorous comparative analysis of sensor performance across transient operational conditions.

Here, we propose a dynamic hysteresis evaluation framework for strain sensors using strain rate as the key parameter, featuring objective and comparable characteristics. By developing a mathematical calculation method to calculate hysteresis errors between sensor outputs and reference signals under dynamic loading, it enables quantitative analysis of dynamic hysteresis effects. This method offers an approach for precisely quantifying sensor errors in complex conditions, enhancing accurate modeling of sensor performance in dynamic measurements.

**Fig. 5.**
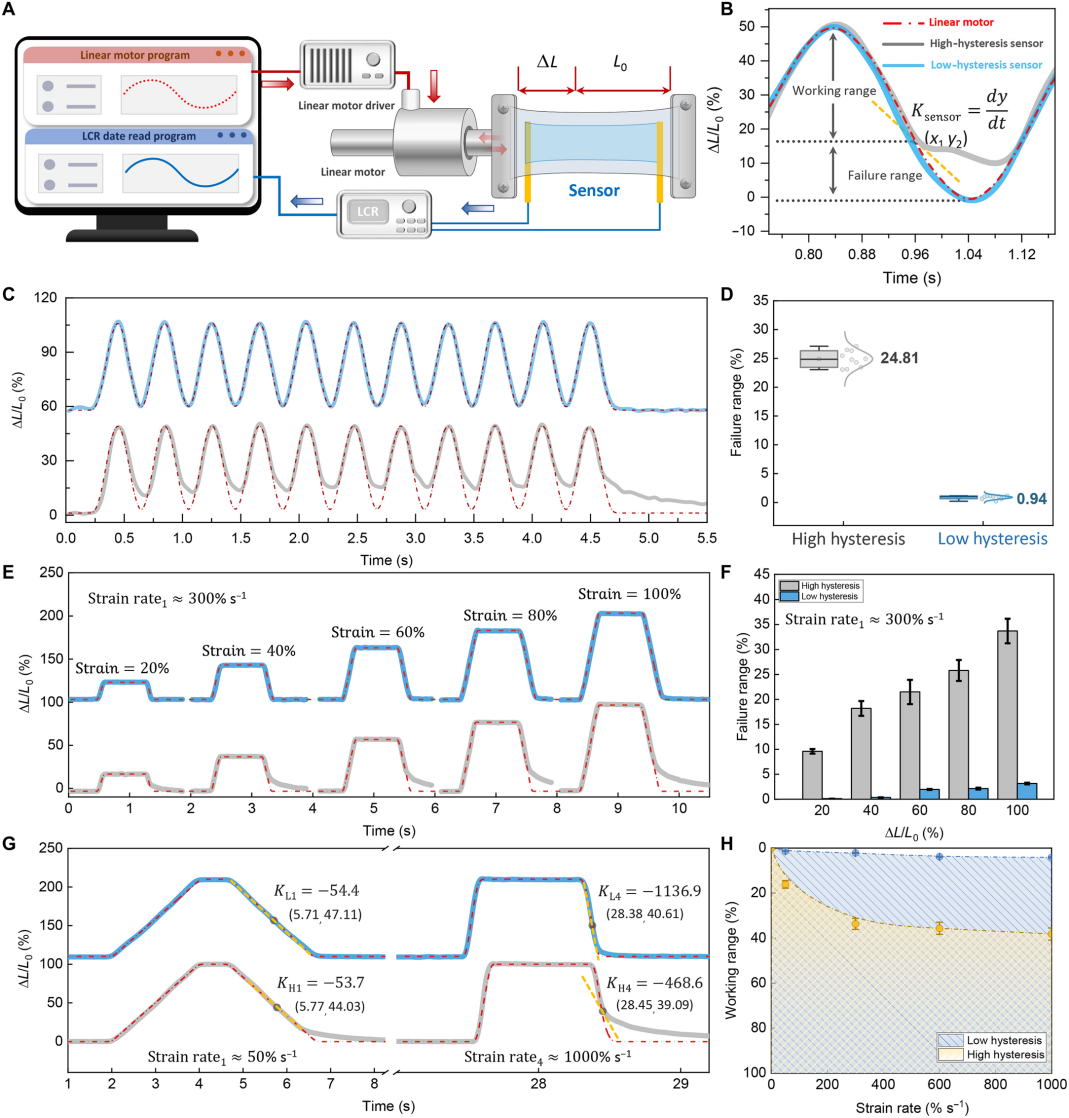
Dynamic hysteresis sensing performance. (**A**) Schematic of the experiment setups for sensors’ dynamic hysteresis monitoring. (**B**) The definition of the failure range of the sensors’ response to a sine strain wave. The response curves (**C**) and failure range (**D**) of high (gray) and low (blue) hysteresis sensors. (**E**) The definition of the failure range of the sensors. High (blue) and low (gray) hysteresis sensors’ response to different strains under the same strain rate. (**F**) The low-hysteresis sensors perform a negligible small failure range compared to high hysteresis sensors. (**G**) High (blue) and low (gray) hysteresis sensors’ response to different strain rates under 100% large strain. (**H**) The effective working range of the sensors. The low-hysteresis sensors remain > 95% effective working range even at extremely high strain rate (1000% s^−1^).

Figure 5A illustrates the architecture of the dynamic hysteresis characteristic testing system. During testing, the host computer sends command signals through a waveform generator to the driving module, which activates the linear motor to generate preset strain patterns. The strain signals captured by the sensor’s sensitive unit undergo analog-to-digital conversion via an inductance, capacitance, and resistance meter (LCR meter) and are simultaneously transmitted back to the data acquisition system, enabling synchronized monitoring and visualization of strain-response signals. The comparative sinusoidal strain response curves in Fig. 5B reveal that the red dashed line represents the theoretical strain waveform output from the host computer, while the blue and gray curves correspond to measured outputs from low-hysteresis and high-hysteresis sensors, respectively. The strain rate is defined as *K*_sensor_

$$K_{\text{sensor}}=\frac{\mathrm{dy}}{\mathrm{dt}}$$ (1)

where *y* = 100% * Δ*L*/*L*_0_.

The equipment strain rate is defined as *K*_strain_

$$K_{\text{strain}}=100\%*\left(v_{\text{Motor}}/L_{0}\right)$$ (2)

where $v_{\text{Motor}}$ is the linear motor extension/retraction speed, which can be set in the device software.

Notably, the low-hysteresis sensor demonstrates exceptional waveform consistency (>99% overlap) with the theoretical profile, whereas the high-hysteresis sensor exhibits substantial phase deviation beyond a 15% strain threshold. This hysteresis phenomenon originates from the dynamic mismatch between sensor response rate (*K*_sensor_) and system loading speed (*K*_strain_), causing complete signal distortion below 15% strain and forming distinct dynamic detection blind zones. The failure threshold is defined as the critical state where sensor strain rate deviation exceeds 10% of the theoretical value, mathematically characterized by 10% relative dynamic hysteresis error in the first derivative of the response curve, with the corresponding strain interval’s lower limit marking the dynamic failure initiation point. The relative dynamic hysteresis error is defined as following

$$\text{Relative dynamic hysteresis error}=\frac{|K_{\text{sensor}}-K_{\text{strain}}|}{K_{\text{strain}}}$$ (3)

Next, we prepared various types of sensors for testing, including a low-hysteresis sensor with Ecoflex encapsulation (encapsulation layer:dielectric layer thickness ratio = 3.2:1); a high-hysteresis sensor with VHB 1 encapsulation (encapsulation layer:dielectric layer thickness ratio = 3.2:1); and a conventional high-hysteresis sensor, designed without an encapsulation structure. Under continuous testing at 2 Hz (approaching the bending frequency of human joints) and 50% peak strain (Fig. 5C), the low-hysteresis sensor demonstrates a 26-fold reduction in dynamic failure range (0 to 0.94%) compared with conventional high-hysteresis devices (0 to 24.81%) as shown in Fig. 5D. Trapezoidal wave loading tests (Fig. 5E) further reveal nonlinear expansion of failure ranges for both device types when strain rates reach 300% s^−1^. Even under extreme 100% strain conditions, the low-hysteresis device maintains a failure range below 5%, notably outperforming the 30% failure range observed in high-hysteresis counterparts (Fig. 5F).

Strain rate sensitivity experiments (Fig. 5G and fig. S10) demonstrate that at 100% peak strain, the low-hysteresis sensor maintains a sub-5% failure range (fig. S11) under ultrahigh strain rate 1000% s^−1^ loading, while high-hysteresis devices exceed 40% failure range. We tested the failure range of the sensors under different strain rates from 50 to 1000% s^−1^. Through further calculations and mathematical fitting, we delineated the effective working ranges of sensors under varying strain rates, thereby enabling the quantification of sensing reliability at different response speeds. As shown in Fig. 5H, the blue-shaded area represents the effective working range of the low-hysteresis sensor, while the yellow zone corresponds to the high-hysteresis sensor. The results demonstrate a contraction in the effective working range with an increasing strain rate. At a strain rate of 1000% s^−1^, the high-hysteresis sensor exhibited a diminished effective working range (40 to 100%), whereas the low-hysteresis sensor maintained more than 95% of its effective working range (4.1 to 100%).

Traditional methods for quantifying sensor hysteresis characteristics exhibit notable limitations. They are primarily confined to quantifying hysteresis effects under specific static strains and constant strain rates. This limitation fundamentally stems from the absence of time-dependent parameters, rendering them ineffective for adapting to the random dynamic working conditions prevalent in practical engineering applications. This work innovatively proposes a strain-rate-parameter-based dynamic quantification method for hysteresis error, which demonstrates notable superiority in characterizing dynamic hysteresis behavior. After systematically acquiring the sensor’s working range data under multigradient strain rates through experimental studies, the constructed mathematical calculation method enables dynamically evaluating the reliability level of randomly varying strain rate and strain amplitude data at any given time point. The establishment of this method provides a theoretical foundation and technical pathway for research on sensor dynamic characteristics.

Our sensor not only exhibits exceptionally low dynamic hysteresis but also imparts several additional advantageous properties to the design of the stretchable heterogeneous encapsulation layered structure. For instance, the robust encapsulation layer effectively protects the device, enabling it to demonstrate high resilience in complex real-world environments. Table S1 provides a performance comparison with state-of-the-art pressure sensors, evaluating key metrics including sensitivity, quasistatic hysteresis, dynamic hysteresis, linearity, sensing range, cyclic loading, and transduction stability (*4*, *45*, *48*–*52*, *70*–*72*). The results indicate that the sensor developed in this work achieves commendable overall properties across these parameters.

### Applications of the sensor for the real-time human-machine interaction system

The implementation of real-time human-machine interface systems necessitates sensing components capable of maintaining precise strain quantification under conditions of rapid dynamic loading and extensive deformation ranges. Our developed sensors demonstrate exceptional suitability for these demanding operational environments through their robust performance characteristics.

**Fig. 6.**
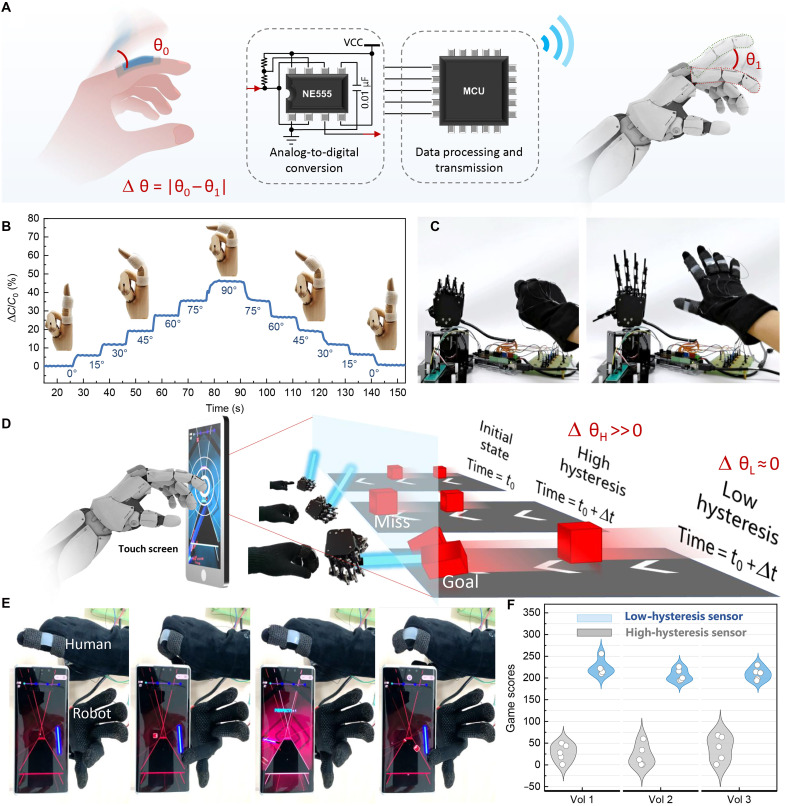
Demonstration of the real-time human-machine collaborative systems. (**A**) Schematic diagram of the bidirectional human-machine interface platform. (**B**) Capacitance variation profiles recorded from sensors affixed to the finger joint of an artificial hand model during flexion-extension maneuvers (finger bending angles measured at 15° increments from 0° to 90°). (**C**) Demonstration of the sensor-integrated glove for human-machine collaborative systems. (**D**) Comparative performance analysis of robotic manipulation in gaming applications using sensors with differential hysteresis characteristics (high- versus low-hysteresis variants). This configuration validates the sensor’s temporal resolution and signal fidelity in dynamic gesture recognition critical for collaborative automation tasks. (**E**) Images of operator gestures and robotic actuation during real-time gaming scenarios. (**F**) Quantitative performance metrics comparing user proficiency with high-hysteresis versus optimized low-hysteresis sensor configurations in dexterity-dependent applications. This systematic evaluation illustrates the efficacy of hysteresis-engineered sensors in maintaining temporal signal consistency and motion tracking precision required for advanced human-machine collaborative systems. MCU, microcontroller unit; VCC, supply voltage powering the NE555 chips.

To validate the technological potential in wearable interactive systems, five isometric sensing units were strategically distributed across a wearable glove apparatus for real-time motion capture applications. As illustrated in Fig. 6A, the capacitive response modulation induced by phalangeal articulation was converted into frequency-modulated pulse waveforms through a multiharmonic oscillation circuit incorporating NE555 timers. Subsequent signal processing via the Arduino Mega 2560 microcontroller unit (MCU) enabled precise actuation control of the robotic manipulator.

The sensing architecture exhibited superior metrological performance across both static and dynamic strain regimes. Quantitative characterization (Fig. 6B) revealed that the low-hysteresis variant achieved exceptional measurement stability during incremental flexion testing (0° to 90° range, 15° intervals) on a prosthetic hand articulation joint. Further resolution analysis using a calibrated rotational stage (14-mm radius) confirmed angular detection limits of 1.2° (fig. S12), satisfying practical implementation requirements.

Real-time synchronization between manual gestures and robotic replication was successfully demonstrated (Fig. 6C). A comparative hysteresis analysis (Fig. 6, D and E) evaluated system performance in time-critical applications through mobile gaming simulations requiring precise temporal coordination. In real-time mobile games, the manipulator’s finger needs to click the screen within a specific time to eliminate blocks and earn points in the game (movie S2). If the robot missed five squares, then the game would end. Sensor signal hysteresis causes the robotic hand’s motion to lag behind human hand movements, creating a temporal delay between actual screen contact and the intended tap timing, resulting in missed squares. The system with VHB 1 encapsulation (with a thickness ratio of 3.2:1) high hysteresis showed a high angle error (Δθ_H_ >> Δθ_L_), which resulted in a high missed target rate compared to the low-hysteresis system. Through the statistical analysis of gaming performance metrics (Fig. 6F), it was revealed that there was a statistically significant advantage (about 200% improvement in game scores) for the optimized sensor configuration. This advantage was attributed to the reduced signal latency and the improved transient response characteristics. These findings substantiate that hysteresis minimization is critical for achieving subsecond latency requirements in human-machine interaction systems.

## DISCUSSION

In conclusion, this work presents a hysteresis-mitigated strain sensing system that uses an innovative interface coordination strategy, guided by the Kelvin-Voigt viscoelastic model. By developing a rational interfacial synergistic architecture, we achieved substantial hysteresis suppression through viscoelastic energy modulation and molecular-scale interlocking enabled by a dual-network deformable adhesive system. This approach effectively integrates heterogeneous elastomer-hydrogel interfaces while maintaining stretchability, allowing for the assembly of low-hysteresis multilayer elastomeric composites. The resulting capacitive strain sensor demonstrates exceptional linearity (*R*^2^ = 0.9998) within a 200% strain range and outstanding dynamic performance, achieving less than 1% hysteresis error (at 100% strain and a strain rate of 50% s^−1^). Our proposed dynamic hysteresis evaluation framework, which accounts for strain-rate dependency and uses a mathematical approach for calculating signal-reference deviations, establishes a standardized methodology for quantifying dynamic errors in complex situations. Enhanced mechanical resilience is evident through a 93.56% consistency across devices and high tolerances for damage, while minimized phase lag and signal distortion improve responsiveness in human-machine interfaces. Robotic validation trials further affirm the operational reliability of our sensors in stochastic dynamic environments, showcasing notable advancements in performance during high-speed manipulation tasks. This work contributes to the field of precision sensing technology by integrating material innovation, interfacial engineering, and dynamic error quantification, thereby offering a transformative paradigm for next-generation soft electronics in robotics, wearables, and industrial automation under challenging dynamic conditions. To enhance comparability across future studies, it is essential to establish standardized testing methodologies and parameters for quantifying hysteresis performance. Specifically, uniform strain ranges and strain rates during testing are critical, yet many existing studies do not specify these factors. Furthermore, the development of precise and versatile testing equipment capable of evaluating sensor hysteresis performance under extreme conditions is paramount. Addressing the challenge of reliably capturing sensing variables and strain rates in high-speed dynamic environments without delays is vital for future advancements.

## MATERIALS AND METHODS

### Materials

All chemicals were used as received unless otherwise specified. SH-PAAm (Shanxi Carryherb Bio Co. Ltd.), poly(vinyl alcohol) (PVA) (molecular weight of 89,000 to 98,000 kDa, 99%; Aladdin), and acrylamide (AAm; 99%; Aladdin) served as the primary hydrogel network components. LiCl (99%; Innochem) functioned as a hygroscopic conductive additive, while glycerin (99%; Aladdin) provided moisture retention and antifreeze properties. Cross-linking was achieved using *N*,*N*-methylenebisacrylamide (MBAA; 99%; Aladdin) and polyethylene glycol diacrylate (PEGDA; Aladdin). The photoinitiator used was 2-hydroxy-4′-(2-hydroxyethoxy)-2-methylpropiophenone (Irgacure 2959, Sigma-Aldrich, 410896). Elastomers included VHB tapes [VHB 4905 (500 μm) and VHB 4920 (400 μm), 3M] and PDMS (KRR 300, Huangzhou Gunei). Surface modification of VHB was performed by spin coating a 10 wt % benzophenone solution in ethanol to enhance interfacial adhesion. A hybrid adhesive was formulated by blending polysiloxane (Sil-Poxy, Smooth-On), 2,2,4-trimethylpentane (Sigma-Aldrich, 360066), and cyanoacrylate (LOCTITE 406, Henkel) in a ratio of 5:4:1 for bonding hydrogel to elastomer components. The stiff backing PET (0.07 mm; Fangcheng Plastic Industry Co. Ltd.) films were used together with cyanoacrylate.

### Preparation of hydrogel precursor solution

A 2.5 wt % solution of SH was prepared by dissolving SH powder in deionized water under continuous stirring at 100 rpm for 1 hour. Subsequently, AAm was added at a concentration of 0.333 g/ml and dissolved via magnetic stirring at 25°C for 30 min. To this solution, LiCl (2 M relative to AAm), glycerin (1:1 w/w with respect to AAm), MBAA (0.4 wt % of AAm), PEGDA (0.42 wt % of AAm), and the photoinitiator Irgacure 2959 (1.1 wt % of AAm) were sequentially added, ensuring complete dissolution after each addition.

For the preparation of the tough hydrogel precursor solution used in the 90° peel strength test, PVA powder (10 wt %) was first dispersed in deionized water and stirred at 100 rpm for 1 hour at room temperature (RT) to avoid aggregation. The dispersion was then transferred to a 90°C oil bath and stirred continuously at the same speed for another hour to achieve complete homogenization. After cooling the solution to RT, AAm was added at 0.333 g/ml and dissolved under stirring at 25°C for 30 min. The same additives—LiCl (2 M relative to AAm), glycerin (1:1 w/w with respect to AAm), MBAA (0.4 wt % of AAm), PEGDA (0.42 wt % of AAm), and Irgacure 2959 (1.1 wt % of AAm)—were then introduced sequentially, ensuring thorough dissolution after each step. Last, the precursor solution was degassed under a nitrogen atmosphere for 1 hour before being cured under ultraviolet (UV) light (365 nm, ZLUVLAMP-50).

### Sensor assembly

Unless otherwise specified, all sensor dielectric layers use VHB 4905. VHB strips (50 mm by 15 mm by 0.5 mm) were coated with benzophenone via spin coating at 500 rpm for 30 s to facilitate interfacial bonding. A PET mold (30 mm–by–5.5 mm opening) was aligned onto the treated VHB surface, followed by injection of the hydrogel precursor solution. UV curing was performed under 365-nm light for 1 hour. The resulting hydrogel-VHB hybrid was encapsulated with elastomer layers (50 mm by 15 mm) using the developed hybrid adhesive. Symmetrical gold electrodes (1.2-mm width) were integrated on both sides through iterative bonding of hydrogel, elastomer, and VHB layers.

### Characterization

Morphological analysis was conducted using SEM (Hitachi, SU1510) on lyophilized hydrogel cross sections. Chemical characterization used Fourier transform infrared spectroscopy (Bruker, VERTEX 80v) and Raman spectroscopy (LabRAM HR Evolution, HORIBA). Mechanical properties were evaluated with a tensile testing machine (Yuelian YL-S71), and dynamic mechanical behavior was examined using a linear motor–based setup (LinMot) with custom clamps. Capacitance measurements were recorded with an LCR meter (Agilent, E4980A), while stress-relaxation behavior was characterized via dynamic mechanical analysis (Q800).

### Finite element simulation of elastomer hysteresis

Finite element analysis was performed in Abaqus 2020 to model stress distribution and deformation in VHB, Ecoflex, and VHB-Ecoflex composites. The models incorporated dimensions of 50 mm by 15 mm by 0.5 mm for VHB and 50 mm by 15 mm by 1.6 mm for Ecoflex, with Young’s moduli of 50 and 100 kPa, respectively, and Poisson’s ratios of 0.49. Material behaviors were derived from experimental stress-relaxation data, assuming perfect interfacial bonding. To evaluate hysteresis, one end of the specimens was fixed, while the other was stretched to 100% strain and subsequently released. The extent of hysteresis was inferred from the slow recovery behavior observed during unloading.
