# Supplementary material for: Hysteresis-free and dynamically resilient strain sensor enabled by interfacial coordination
Source: Sci Adv. 2026 Jan 1;12(1):eaea2450. doi: 10.1126/sciadv.aea2450 (PMC12757069; doi:10.1126/sciadv.aea2450)
Supplement: Supplementary file 1 — Figs. S1 to S12 Supplementary Text Table S1 Legends for movies S1 and S2 [file sciadv.aea2450_sm.pdf]

Science Advances
AAAS

Supplementary Materials for

Hysteresis-free and dynamically resilient strain sensor enabled by interfacial coordination

Jiang He *et al.*

Corresponding author: Caofeng Pan, pancaofeng@buaa.edu.cn; Rongrong Bao, baorongrong@buaa.edu.cn; Chuan Fei Guo, guocf@sustech.edu.cn; Wenchao Gao, gaowenchao@binn.cas.cn

Sci. Adv. **12**, eaea2450 (2026)
DOI: 10.1126/sciadv.aea2450

The PDF file includes:

Figs. S1 to S12
Supplementary Text
Table S1
Legends for movies S1 and S2

Other Supplementary Material for this manuscript includes the following:

Movies S1 and S2

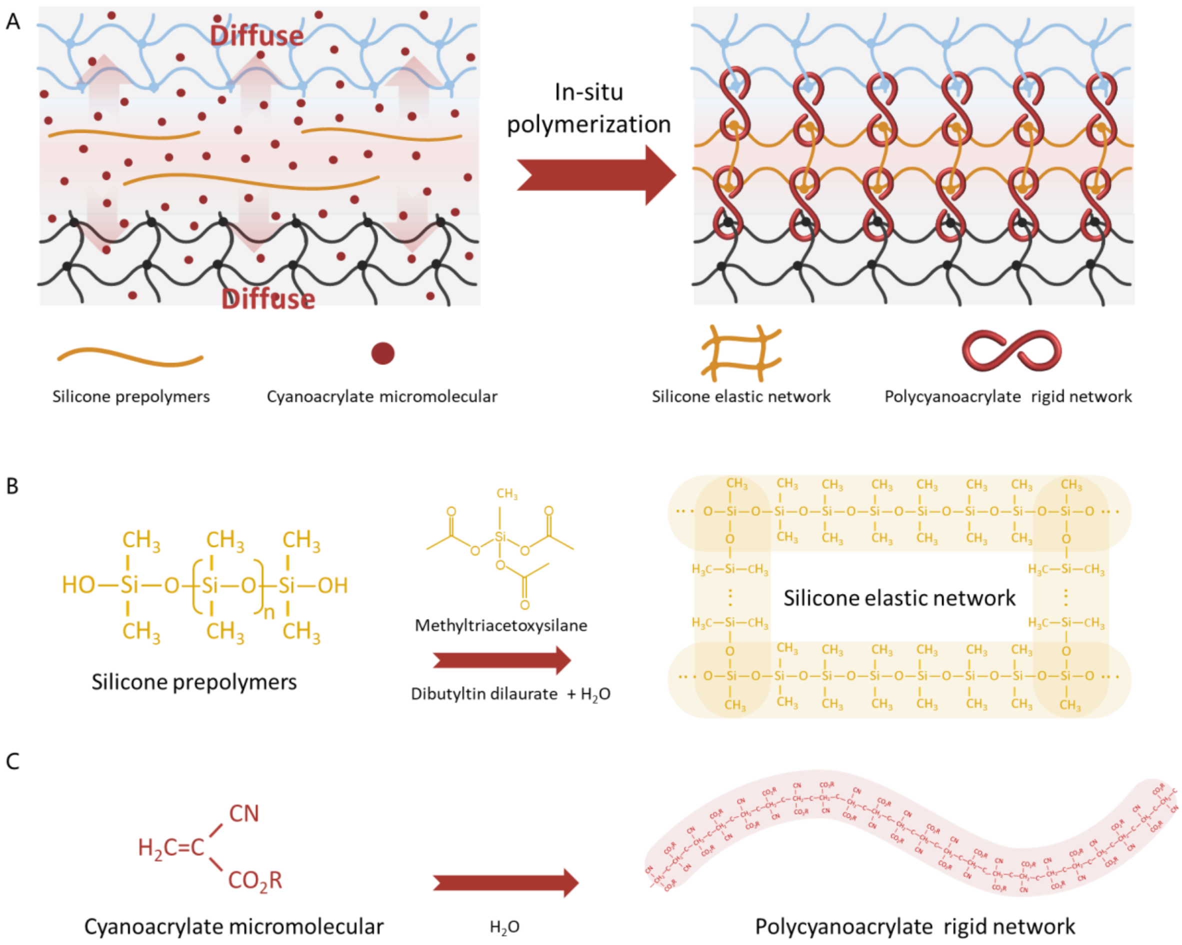

Fig. S1. The formation mechanism of the dual-network of the stretchable adhesive. (A)The in-situ polymerization process of dual-network stretchable adhesive. The flexible network consists of silicone molecular chains that transmit strain through high elasticity; the rigid network comprises polycyanoacrylate chains, where cyanoacrylate monomers directionally penetrate bonded materials (hydrogels and elastomers) during adhesion and polymerize in-situ to form interpenetrating entangled networks. (B) The reaction polymerization mechanism of a flexible network. The crosslinking process of condensation-curable silicone rubber focuses on the formation of covalent Si-O-Si bonds between siloxane chains. It depends on silicone prepolymers containing hydroxyl groups (-Si-OH), which undergo a condensation reaction with crosslinkers (multifunctional silanes, e.g., methyltriacetoxysilane) in the presence of catalysts (e.g., dibutyltin dilaurate). (C) The reaction polymerization mechanism of a rigid network. The polymerization of cyanoacrylate (e.g., ethyl α-cyanoacrylate, the main component of instant adhesives like "super glue") is an anionic reaction, with water molecules as the key initiator. The process has three steps: First, the hydroxyl group in water acts as a nucleophile, attacking the electron-deficient carbon in the cyanoacrylate double bond (rendered electron-poor by the electron-withdrawing cyano and ester groups) to form a negatively charged active alkoxide ion intermediate (initiation stage). Next, this intermediate continuously attacks the double bonds of other monomers, extending the polymer chain via nucleophilic addition (propagation stage). Finally, trace proton donors (e.g., water, alcohols) in the system neutralize the negative charge at the end of the active chain, terminating the reaction and producing high-molecular-weight polycyanoacrylate.

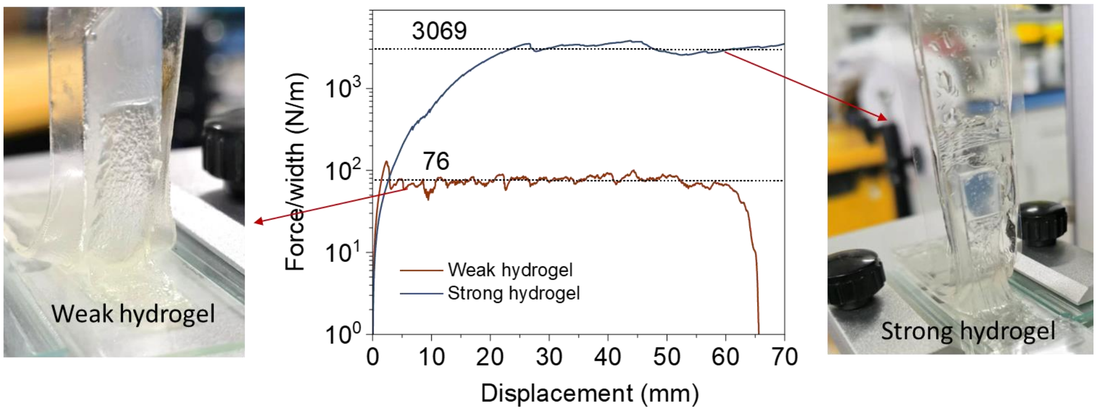

Fig. S2. Measurements of peeling force per unit width for weak and strong hydrogel-VHB hybrid bonding interfaces.

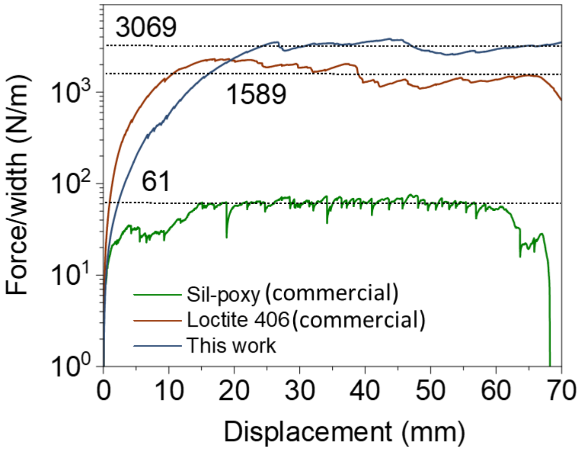

Fig. S3. The performance comparison between commercial adhesives and the adhesives in this work.

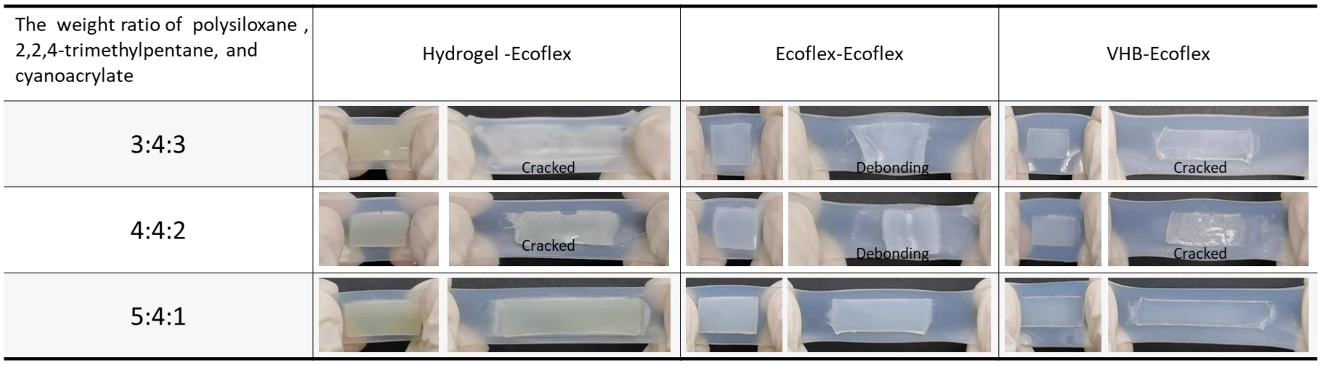

Fig. S4. Effect of adhesive composition ratio on performance. Experimental results demonstrate that unbalanced formulations (3:4:3 and 4:4:2) induce two typical failure modes: (1) interface debonding caused by component incompatibility, and (2) micro-crack nucleation and propagation under tensile stress due to excessive brittle phase content. Both failure mechanisms lead to a non-uniform strain field distribution at the interfacial region, resulting in significant interface energy dissipation.

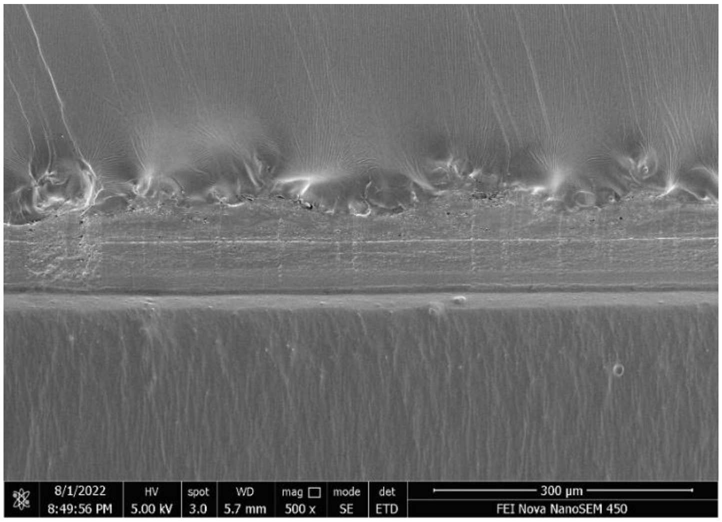

Fig. S5. The cross-sectional SEM images of the multilayer hydrogel–elastomer bonding hybrids. The image revealed the seamless microscopic integration structure of the multilayer hydrogel–elastomer hybrid materials.

Fabrication and characterization of the composites of hydrogels and elastomers

The fabrication process of the hydrogel–dielectric composite structure is schematically illustrated in Fig. S6-A. Viscoelastic polyacrylate elastomers (VHB) were selected as dielectric substrates, owing to their high deformability, intrinsic adhesiveness, and enhanced permittivity. The first step involved surface modification of VHB membranes via ethanol-based benzophenone treatment (10 wt%), which mitigated oxygen inhibition and facilitated subsequent free radical polymerization and interfacial covalent bonding.

A laser-patterned polyethylene terephthalate (PET) mask featuring microscale "Science" motifs was precisely aligned with the activated VHB surface. The SH-PAAm hydrogel precursor solution was then deposited into the patterned mold, followed by UV irradiation (λ = 365 nm, 1 h) under a glass cover. Post-fabrication analysis confirmed the successful formation of well-defined microscale hydrogel structures exhibiting strong interfacial adhesion to the VHB substrate, as evidenced by the preserved structural integrity under tensile deformation (Fig. S6-B). This interfacial robustness stemmed from synergistic molecular interactions at the hydrogel–elastomer interface, including benzophenone-mediated graft polymerization and topological entanglement.

Cross-sectional scanning electron microscopy (Fig. S6-C) confirmed seamless integration between hydrogel and elastomer layers, with no observable interfacial voids. The characteristic micro-wrinkled morphology of the VHB further contributed to its strain-accommodation capability. Complementary spectroscopic characterization validated successful hydrogel synthesis: Raman spectral (Fig. S6-D) and FT-IR (Fig. S6-E) analysis demonstrated characteristic peak inversion phenomena, manifesting as attenuation of =C-H bending mode (998 cm^{-1}), C=C stretching vibrations (1635 cm^{-1}) concurrent with intensification of C-H vibrational modes (2930 cm^{-1}), thereby confirming successful crosslinking progression on VHB surfaces.

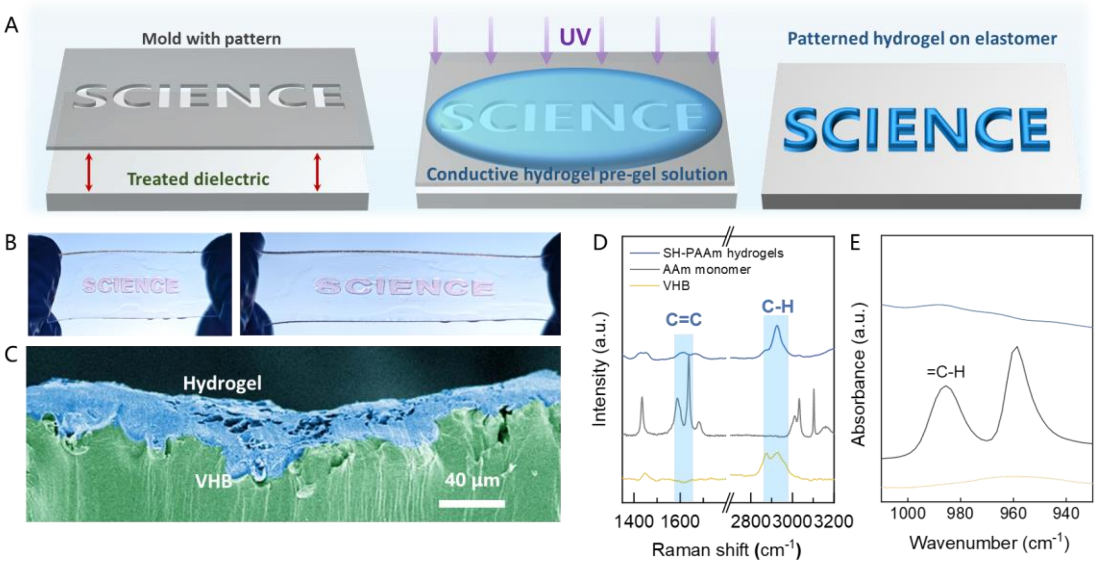

Fig. S6. Fabrication and characterization of the composite of SH-PAAm hydrogels and elastomers. **(A)** Schematic illustration of the fabrication process of patterned hydrogels-dielectric hybrids. (**B)** The photographs of patterned SH-PAAm hydrogels on the surface of VHB at the original and stretching state. (**C)** The cross-section morphology of SEM of SH-PAAm hydrogels bonding with the dielectric layer of VHB. (**D)** Raman spectrum of the C=C and C-H in SH-PAAm hydrogels, AAm monomer, and pure VHB. (**E)** FT-IR spectrum of the =C-H in SH-PAAm hydrogels, AAm monomer, and pure VHB.

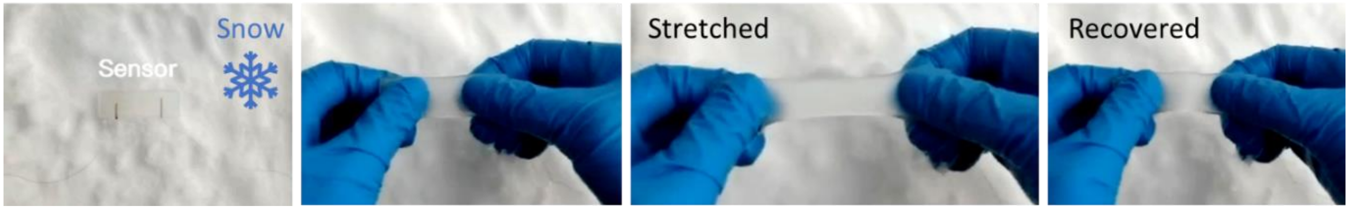

Fig. S7. Demonstration of the anti-freezing performance of the sensor. As shown in the images, the sensor maintains good elasticity even at -10°C.

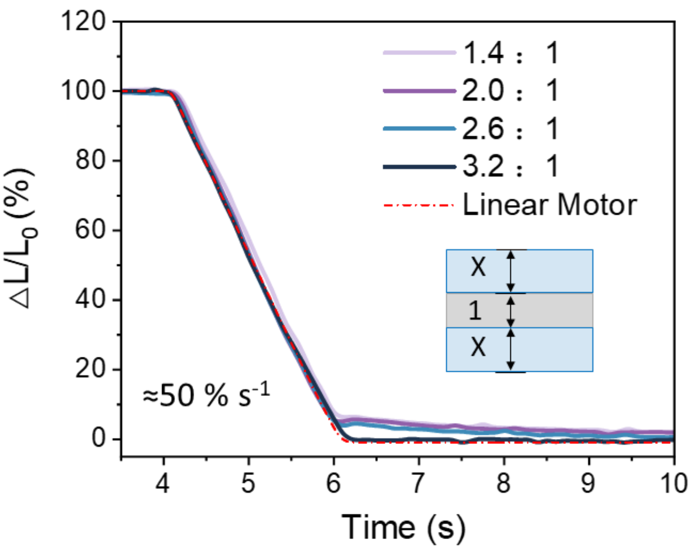

Fig. S8. The ratio of thicknesses between the different layers impacts the hysteresis of the composite stack. The thickness of the dielectric layer is fixed at 1, where X represents the thickness of the encapsulation layer.

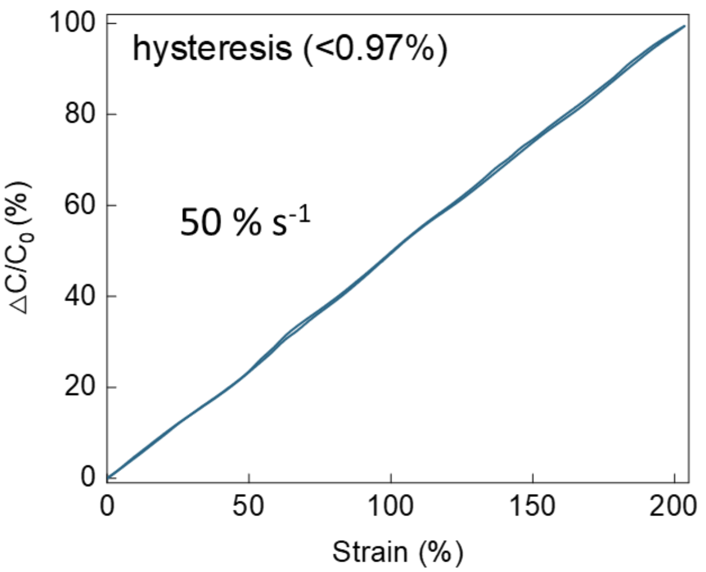

Fig. S9. Capacitance variation characteristics of the low-hysteresis system under cyclic stress loading-unloading cycles.

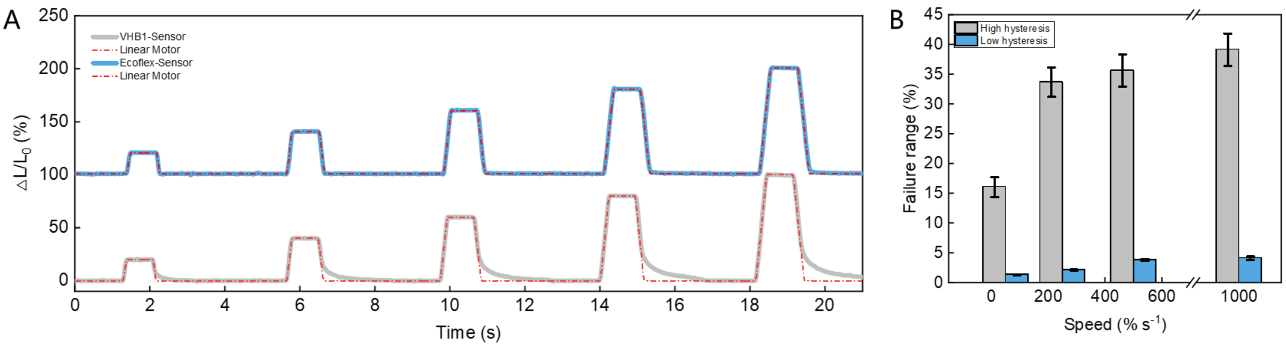

Fig. S10. Dynamic hysteresis sensing performance. (A) High (gray) and low (blue) hysteresis sensors' response to different strains under a large strain rate of 300% s^{-1}. **(B)** The failure range of sensors at different strain rates.

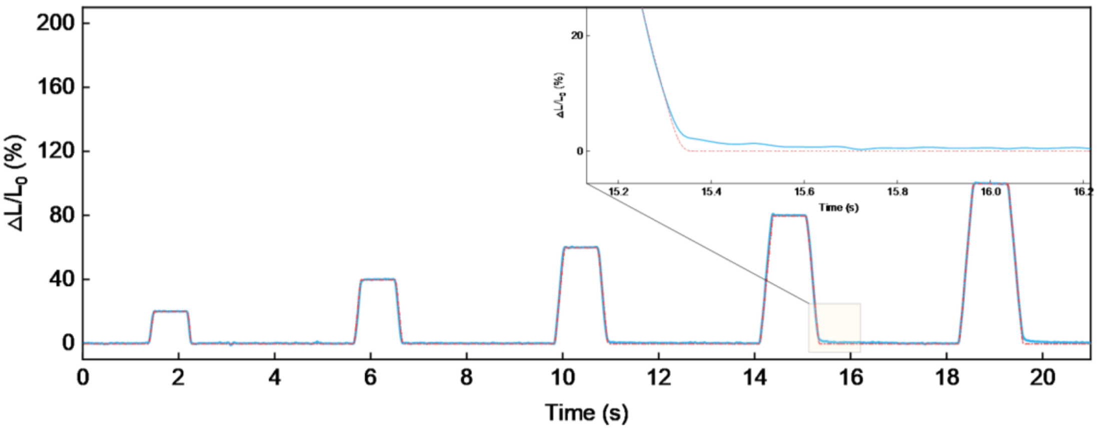

Fig. S11. Response of low-hysteresis sensors to varying strain under a high strain rate of 300% s^{-1}. The magnified image section reveals minor signal hysteresis at strains below 5%.

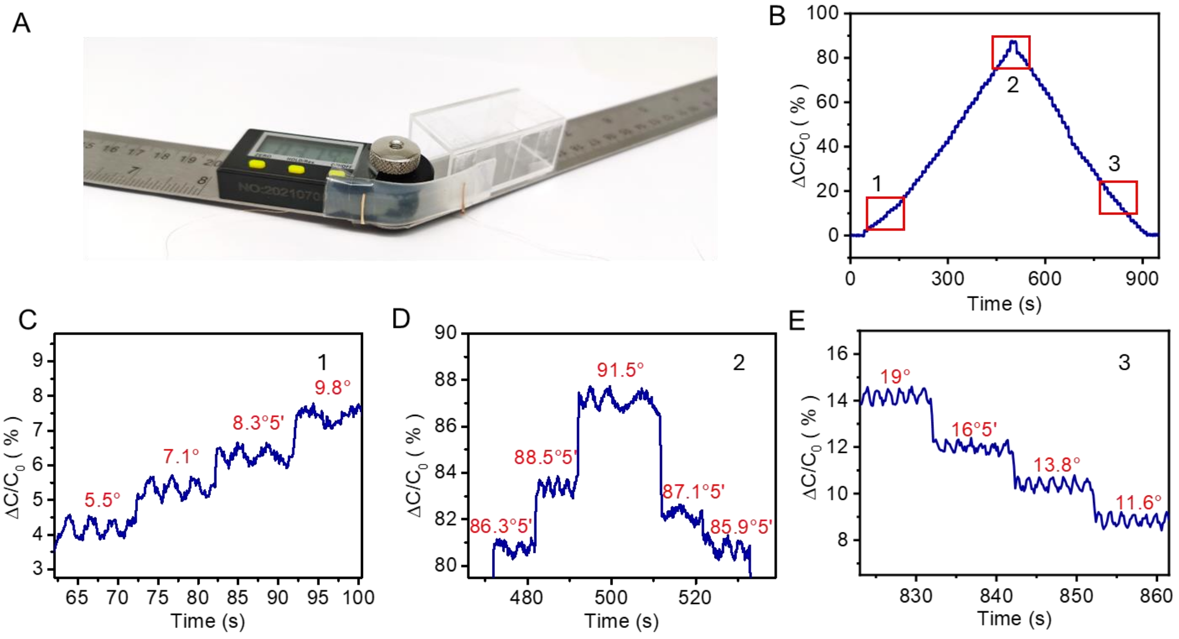

Fig. S12. Capacitance variation profiles acquired from goniometric-integrated sensors during controlled angular displacement from 0° to 90°. (A) Photo of goniometric-integrated sensors. **(B)** Capacitance variation profiles recorded from sensors. **(C), (D)** and **(E)** are the locally enlarged views of the capacitance variation curve.

Table S1. Performance comparison with state-of-the-art strain sensors

Materials	Sensitivity/Type	Quasi-static hysteresis	Dynamic hysteresis	Linearity	Sensing range	Cyclic loading	Transduction stability	Ref.
Hydrogel(SH- AAM)/VHB/Ecoflex	GF=0.49/Capacitive	0.97% (200% strain, 50 % s^{-1} strain rate)	0.94% (@50% s^{-1}) 4.15% (@1000% s^{-1})	0.9998	0%-204%	10064 (@50%)	withstand low temperatures, impact, bending, pressure, and twisting	**This work**
carbon black-filled elastomer	GF=0.86/Capacitive	—	—	0.9995	0%-200%	10000 (@300%)	—	(*51*)
liquid metal/PDMS	GF=-0.304/Capacitive	1% (50% strain, 0.28 % s^{-1} strain rate)	—	0.986	0%-100%	300 (@30%)	—	(*50*)
liquid metal/Ecoflex	GF=4.91/Resistive	6.79% (320% strain, 15 % s^{-1} strain rate)	—	—	0%-320%	500 (@100%)	—	(*70*)
PVA−PEDOT/PSS hydrogel	GF=0.7/Resistive	—	—	—	0%-500%	1000 (@100%)	withstand bending, twisting, and pressure	(*49*)
PEDOT:PSS/Ecoflex	GF=0.49/Resistive	6.79% (300% strain, — strain rate)	—	0.98	0%-300%	2000 (@85%)	withstand twisting and pressure	(*71*)
Carbon fiber polymer composites/PU films	GF=9400/Resistive	≈16.8% (1.50% strain, 0.67 % s^{-1} strain rate)	—	0.98	< 5 %	1000	withstand impact, bending, twisting, and pressure	(*73*)
3D mesostructures	GF=−0.30/Capacitive	2.8% (150% strain, 80 % s^{-1} strain rate)	—	—	0%-400%	10000(@100%)	—	(*45*)
TA@HAP NWs-PVA(EG/W) hydrogel	GF=2.84/Resistive	2.4% (300% strain, — strain rate)	—	0.99003	0%-300%	300 (@50%)	withstand low temperature	(*72*)
gradient stiffness sliding structure	GF=9.1×10^6/Capacitive	—	—	0.9997	0%-50%	2000 (@40%)	—	(*52*)
wireless and suturable fibre strain-sensing system	GF≈6/Capacitive	≈12.4% (30% strain, 0.25 % s^{-1} strain rate)	—	—	0%-50%	2000 (@10%)	—	(*48*)

Movies S1 **Finite element simulation.**

Movies S2 **Demonstration of a low-hysteresis sensor in gaming applications.**
